# Polyfunctional T Cell Responses in Children in Early Stages of Chronic *Trypanosoma cruzi* Infection Contrast with Monofunctional Responses of Long-term Infected Adults

**DOI:** 10.1371/journal.pntd.0002575

**Published:** 2013-12-12

**Authors:** María C. Albareda, Ana M. De Rissio, Gonzalo Tomas, Alicia Serjan, María G. Alvarez, Rodolfo Viotti, Laura E. Fichera, Mónica I. Esteva, Daniel Potente, Alejandro Armenti, Rick L. Tarleton, Susana A. Laucella

**Affiliations:** 1 Instituto Nacional de Parasitología Dr. M. Fatala Chaben, Buenos Aires, Argentina; 2 Hospital Interzonal General de Agudos Eva Perón, Buenos Aires, Argentina; 3 Hospital Fernandez, Buenos Aires, Argentina; 4 Center for Tropical and Emerging Global Diseases, Athens, Georgia, United States of America; Universidad Autónoma de Yucatán, Mexico

## Abstract

**Background:**

Adults with chronic *Trypanosoma cruzi* exhibit a poorly functional T cell compartment, characterized by monofunctional (IFN-γ-only secreting) parasite-specific T cells and increased levels of terminally differentiated T cells. It is possible that persistent infection and/or sustained exposure to parasites antigens may lead to a progressive loss of function of the immune T cells.

**Methodology/Principal Findings:**

To test this hypothesis, the quality and magnitude of *T. cruzi*-specific T cell responses were evaluated in *T. cruzi*-infected children and compared with long-term *T. cruzi*-infected adults with no evidence of heart failure. The phenotype of CD4^+^ T cells was also assessed in *T. cruzi*-infected children and uninfected controls. Simultaneous secretion of IFN-γ and IL-2 measured by ELISPOT assays in response to *T. cruzi* antigens was prevalent among *T. cruzi*-infected children. Flow cytometric analysis of co-expression profiles of CD4^+^ T cells with the ability to produce IFN-γ, TNF-α, or to express the co-stimulatory molecule CD154 in response to *T. cruzi* showed polyfunctional T cell responses in most *T. cruzi*-infected children. Monofunctional T cell responses and an absence of CD4^+^TNF-α^+^-secreting T cells were observed in *T. cruzi*-infected adults. A relatively high degree of activation and differentiation of CD4^+^ T cells was evident in *T. cruzi*-infected children.

**Conclusions/Significance:**

Our observations are compatible with our initial hypothesis that persistent *T. cruzi* infection promotes eventual exhaustion of immune system, which might contribute to disease progression in long-term infected subjects.

## Author Summary

Chagas disease is a neglected tropical disease affecting approximately 10 million people in the world. As a consequence of migration flows, the disease has also become established in non-endemic countries. Previous studies have demonstrated that *Trypanosoma cruzi*-specific T cells inversely correlates with the severity of cardiac disease in the chronic phase of the infection, suggesting that the immune system becomes exhausted overtime. To test this hypothesis, the quality and magnitude of *T. cruzi*-specific T cell responses were measured in *T. cruzi*-infected children – who are presumed to have shorter-term infections - and compared with long-term *T. cruzi*-infected adults. The activation status of total T cells in *T. cruzi*-infected children was also evaluated. *T. cruzi*-infected children exhibit a more robust, and more highly functional parasite specific T cell responses compared to *T. cruzi*-infected adults. In spite of a more functional immune profile, *T. cruzi*-infected children have a heightened state of immune activation. These observations are compatible with the initial hypothesis that T cell responses specific for *T. cruzi* become exhausted overtime. The impairment in T cell responses might contribute to disease progression in long-term infected subjects.

## Introduction

Chagas disease, a neglected tropical disease affecting approximately 10 million people from south of the United States to Mexico and Central and South America [1], is caused by the protozoan parasite *Trypanosoma cruzi*. As a consequence of migration flows, the disease has been also become established in non-endemic countries [2].

*T. cruzi* frequently results in the development of cardiomyopathy, generally many years after the initial infection. Three factors are likely associated with the development of severe disease: parasite burden; the effectiveness of the host immune response in controlling parasites in specific tissues, and the effectiveness of the host immune response in limiting peripheral damage [3], [4]. Chronic infections in general are associated with a progressive loss of pathogen-specific T cell function known as immune exhaustion [5], [6]. We have previously shown that adults with chronic *T. cruzi*-infections exhibit a prevailing profile of parasite-specific Interferon (IFN)-γ only secreting T cells [7], associated with long-term antigen persistence and exhausted T cells [5]. The frequencies of *T. cruzi*-specific T cells were also found to inversely correlate with the severity of chronic Chagas disease [7], [8].

Although, *T. cruzi*-infected children are likely to have shorter-term infections than most adults, the overall CD8 T cell compartment in children in the early phase of chronic *T. cruzi* infection exhibits decreased levels of naïve T cells and increased levels of terminally differentiated antigen-experienced T cells [9]. Other studies have suggested that *T. cruzi*-infected children have a mixed T cell profile with the production of IFN-γ and IL-4 [10], [11]. However, a comprehensive analysis of the ability of *T. cruzi* specific T cells to co-express multiple functions has not been performed.

In order to examine the progression of immune exhaustion in chronic *T. cruzi* infection, we have assessed the quantitative and qualitative attributes of *T. cruzi*-specific T cell responses in early stages of Chagas disease in children in comparison to *T. cruzi*-infected adults. The degree of activation, differentiation and antigen exposure of total CD4^+^ T cells was also evaluated in *T. cruzi*-infected children. The primary finding is that *T. cruzi*-infected children have a higher frequency of polyfunctional and more robust T cell responses specific for *T. cruzi* compared to *T. cruzi*-infected adults, and a heightened state of immune activation of CD4^+^ T cells.

## Materials and Methods

### Ethics statement

This protocol was approved by the Institutional Review Boards of the Hospital Interzonal General de Agudos Eva Perón, and the Centro Nacional de Genética, Buenos Aires, Argentina. Informed written consent was obtained from adult subjects and the parents of all children included in this study.

### Study subjects

Five to 16-year old children and 29 to 63 year-old adults were enrolled at the Instituto Nacional de Parasitología Dr. Mario Fatala Chaben and at the Hospital Interzonal General de Agudos Eva Perón, (Buenos Aires, Argentina). *T. cruzi* infection was determined by indirect immunofluorescence, haemagglutination and ELISA assays [12]. Subjects positive on at least two of these tests were considered to be infected. All infected children were in the early chronic phase of *T. cruzi* infection. Age- and sex-matched children with negative serological findings were recruited as uninfected controls. *T. cruzi*-infected subjects were classified according to a modified version of the Kuschnir grading system [13], [14]. Most subjects were in the indeterminate phase of the infection (Group 0; positive serology and normal findings on electrocardiogaphy, chest radiography and echocardiography), while 3 subjects (2 adults and one child) belong to the Group 1 group (positive serology, abnormal findings on electrocardiography and normal chest radiography and echocardiography). All participants of this study had not received etiological treatment at the time of sampling and started drug therapy with benznidazole after sampling.

Children and adults with any impaired health condition such as severe nephropathy, liver disease, severe neuropathy, severe anaemia or homeopathy were excluded from the study. Data on the number, sex, age, clinical and epidemiologic features of the subjects included in this study are summarized in Table 1.

**Table 1:** Characteristics of study population.

Clinical group^A^^,^ B	n	Electrocardiographic findings^C^	Number of subjects born in endemic areas	Years of residence in endemic areas,(median) range	Age range (median), years	Gender	
						Male	Female
Seropositive children	49	RBBB (G1)	21	0–12 (0)	5–16 (11)	24	28
		9 ANEA^D^					
Seropositive adults	17	2 PP(G1)	17	1–59 (15)^E^^,^ F	29–63 (52)	10	7
Seronegative children	35	2 ANE	8	0–11 (0)	6–14 (10)	15	20
Seronegative adults	7		3	0–27 (0)	24–60 (51)	4	3

Note.

A All children were born from *T. cruzi*-infected mothers.

B All children and adult subjects were living in Buenos Aires (non-endemic) by the time of the study.

C Number of patients presenting electrocardiographic alterations.

D P<0.05, seropositive children born in areas endemic for *T. cruzi* infection vs. seropositive and seronegative children born in non-endemic areas by Fisher' s exact test.

E P<0.001 vs. seropositive and seronegative children, by Kruskal-Wallis test.

F P<0.05 vs. seronegative adults, by Kruskal-Wallis test.

ANE, abnormal findings in electrocardiography not relevant to Chagas disease; RBBB, right bundle branch block; PP, permanent pacemaker; G1, Group 1 of the Kuschnir grading system.

### Collection of peripheral blood mononuclear cells (PBMC) and sera

Approximately 10 mL of blood were drawn by venipuncture into heparinized tubes (Vacutainer; BD Biosciences). PBMC were isolated by density gradient centrifugation on Ficoll-Hypaque (Amersham) and were cryopreserved for later analysis. Additional 2 mL of blood were allowed to coagulate at 37°C and centrifuged at 1000 *g* for 15 min for sera separation.

### Antigens

Protein lysate from *T. cruzi* amastigotes was obtained by freeze/thaw cycles followed by sonication as previously reported [8].Tetanol Pur (Novartis, Germany) was used as source of tetanus toxoid.

HLA-A01, A02, A03, A24 and B44-supertype binding epitopes encoded by the *trans*-sialidase gene family of *T. cruzi* and peptides derived from Influenza (Flu) virus with high binding-affinity for the common class I HLA-supertypes A01, A02 and A03 were synthesized at the University of Georgia Molecular Genetics Instrumentation Facility (Athens, USA).

### IFN-γ and IL-2 enzyme-linked immunosorbent spot (ELISPOT) assays

The number of *T. cruzi* antigen-responsive IFN-γ- and IL-2-secreting T cells was determined by ex vivo ELISPOT using commercial kits (BD Biosciences), as described elsewhere [7], [8], [15]. Cryopreserved PBMC were seeded in triplicate wells, at a concentration of 4×10^5^ cells/well, and stimulated with *T. cruzi* lysate (10 µg/mL) or with peptide pools from the *trans*-sialidase protein family (7–13 peptides per pool, 5 µg/ml/peptide). For controls, PBMCs were incubated with either 20 ng/mL phorbol 12-myristate 13-acetate (PMA, Sigma) plus 500 ng/mL ionomycin (Sigma), Flu virus (3 peptides per pool, 5 µg/ml/peptide), tetanus toxoid (4 I.U./ml) or media alone (unstimulated control) for 16–20 hr at 37°C and 5% CO_2_. Spot forming cells (SFC) were automatically enumerated using ImmunoSpot analyzer (CTL). The mean number of spots in triplicate wells was obtained for each condition, and the number of specific IFN-γ and IL-2-secreting T cells was calculated by subtracting the value of the wells containing media alone from the antigen-stimulated spot count. Responses were considered significant if a minimum of 10 spots/4×10^5^ PBMC were present per well, and additionally, this number was at least twice the value of wells with media alone [8].

### Whole blood intracellular and surface staining assays

For polyfunctional analysis, 200 µL heparinized blood, half diluted in RPMI, were incubated with *T. cruzi* lysate in the presence of anti-CD28 and anti-CD49d antibodies (1 µg/ml; BD Pharmingen), for 16–20 h at 37°C. Ten µg/ml brefeldin A (Sigma) were added for the last 5 h of incubation, as previously described [16]. Blood incubated with no antigen served as a negative control (unstimulated control), while blood incubated with Staphylococcal enterotoxin B (1 µg/ml; Sigma-Aldrich) served as a positive control. Twenty mM EDTA was added for 15 min. Cells were then stained with anti-human CD4-peridinin chlorophyll protein (PerCP) ensued by red cell lysis and white cell fixation in FACS Lysing Solution (Pharmingen). This was followed by fixation and permeabilization with Cytofix/Cytoperm solution (Pharmingen) according to manufacturer's instructions. Cells were stained with a combination of monoclonal antibodies specific for IFN-γ [phycoerythrin (PE)], tumor necrosis factor (TNF)-α [allophycocyanin (APC)] and CD154 (CD40L) [fluorescein isothiocyanate (FITC)], all from BD Bioscience. Since CD154 is only transiently expressed on the cell surface [17], intracellular expression of CD154 was measured, as previously described [18]. In order to confirm that cytokine/co-stimulation expression was derived from T cells, anti-human CD3-FITC or CD3-PerCP was added in polyfunctional staining assays in combination with CD4, IFN-γ and TNF-α or CD4, IFN-γ and CD154, respectively.

Typically, 500,000 lymphocytes were acquired on a FACScalibur (Becton Dickinson Immunocytometry Systems) and analyzed using FlowJo software (TreeStar, Inc.) Lymphocytes were identified based on their scatter patterns and CD4 expression for the combination of IFN-γ, TNF-α and CD154; and based on scatter patterns as well as CD3 and CD4 expression for the combination of IFN-γ and TNF-α or IFN-γ and CD154. Boolean combination gating was then performed to calculate the frequencies of expression profiles corresponding to the seven different combinations of functions by using the FlowJo software. After subtracting the background values, the proportions of the different subsets were expressed as percentages of total cytokine or CD154-positive cells. Responses to the *T. cruzi* lysate were considered positive, for any particular subset, if the frequency of cytokine/CD154-positive T cells was threefold higher than the frequency in medium alone and above 0.07% of total CD4^+^ T cells, since the limit of detection was set at 0.01%.

### Whole blood surface staining assays

For phenotypic analysis of total CD4^+^ T cells, 50 µL of whole blood were incubated with different combinations of CD4 (PerCP), CD45RA (FITC or APC), CD27 (PE), CD28 (PE), CD127 (PE), HLA-DR (FITC) and KLRG1 (APC) monoclonal antibodies (BD Biosciences), followed by red cell lysis and white cell fixation in FACS Lysing Solution (Pharmingen). Typically, 500,000 lymphocytes were acquired on the FACScalibur (Becton Dickinson Immunocytometry Systems) and analyzed using FlowJo software (TreeStar, Inc.)

### Statistical analysis

Demographic and clinical characteristics of *T. cruzi*-infected subjects included in this study were summarized using range and median and compared with healthy participants using Kruskal-Wallis test with Dunn correction and Fisher's exact tests for numerical and categorical variables, respectively. Comparisons of the frequencies of responders in the different categories of ELISPOT responses among *T. cruzi*-infected children and between *T. cruzi*-infected children and the uninfected group were evaluated by use of the chi 2 test and Fisher's exact test. To compare the number of spots,as well as the magnitude of CD4^+^ T cells responses among triple, double or single functional profiles, the Kruskal-Wallis nonparametric analysis with Dunn correction was used. The percentages of responding CD4^+^ T cells for each cytokine within and among the children and adult groups were compared by the Kruskal-Wallis nonparametric analysis with Dunn correction and Mann-Whitney U test, respectively. T cell phenotypes in *T. cruzi*-infected children and uninfected controls were compared applying the Mann-Whitney U-test. Differences were considered to be statistically significant at *P*<0.05.

## Results

### Clinical and demographic characteristics of study population

The main characteristics of the entire study population are summarized in Table 1. *T. cruzi*-infected and uninfected children enrolled in the study were all born from mothers with positive serology for *T. cruzi* infection and, at the time of the study all children were living in Buenos Aires, where *T. cruzi* infection is not endemic. *T. cruzi*-infected and uninfected groups of children included individuals born in areas endemic for *T. cruzi* infection and individuals born in non-endemic areas. Seropositive children who had not lived in endemic areas are presumed to have been infected congenitally. Only one child out of the fifty *T. cruzi*-infected children showed a right bundle branch block that is among the electrocardiographic alterations related to Chagas disease. Abnormal findings in the electrocardiography not related to Chagas disease, including sinusal tachycardia, incomplete right bundle branch block and premature atrial contraction [19], were more frequent among *T. cruzi*-infected children born in areas endemic for *T. cruzi* infection than those born in non-endemic areas (Table 1). The presence of these electrocardiographic alterations were not correlated with the age or gender of children. All *T. cruzi*-infected adults were born in areas endemic for *T. cruzi* infection but have lived in Buenos Aires for more than 15 years, in average. Two adult patients belonged to the G1 clinical stage of the Kuschnir classification [13], showing left anterior fascicular block while the remaining 8 subjects belong to the G0 group.

### Frequencies of cytokine-producing cells specific for *T. cruzi* in children in early stages of Chagas disease

We have previously reported an inverse correlation between the frequency of IFN-γ-producing T cells responsive to *T. cruzi* antigens and disease severity in chronically infected adults [7], [8]. In this study, the magnitude of *T. cruzi*-specific T cell responses was assessed in 17 *T. cruzi*-infected children by measuring the total frequency of IFN-γ- and IL-2-producing cells by ELISPOT assays after stimulation of PBMC with *T. cruzi* antigens. Since we had shown that in chronically *T. cruzi*-infected humans, the frequency of T cells specific for class-I-restricted *T. cruzi* epitopes (CD8^+^ epitopes) [7], [8] or *T. cruzi*–derived recombinant proteins [20] is low to be consistently detected, an amastigote lysate preparation was the primary antigen stimulus for ELISPOT assays.

**Figure 1:**
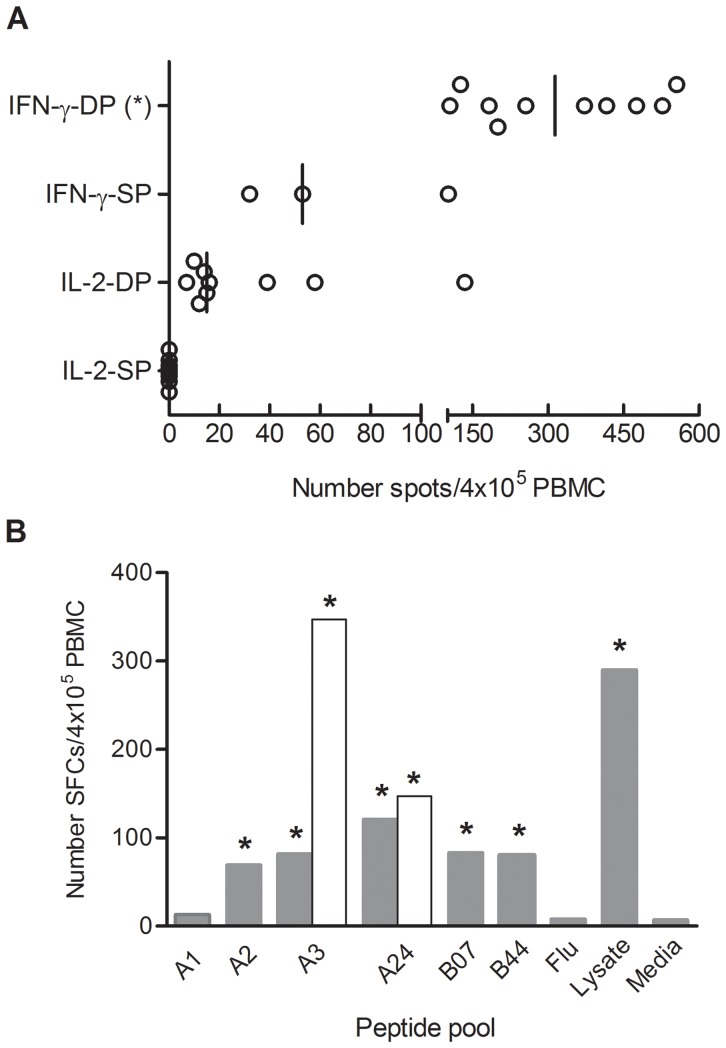
IFN-γ and IL-2-secreting cells in response to *Trypanosoma cruzi* antigens in *T. cruzi*-infected children. PBMC from *T. cruzi*-infected children were seeded at 4×10^5^ cells/well and stimulated with a *T. cruzi* lysate from the Brazil strain, class-I-restricted HLA-A01, HLA-A02, HLA-A03, HLA-A24, HLA-B07 and HLA-B44 supertype *trans*-sialidase peptide pools, class-I-restricted Flu-derived peptides or media alone for 16–20 h. (A) T cell responses to a *T. cruzi* lysate preparation. Each circle represents the mean spot number of triplicate wells for each patient with positive ELISPOT responses out of seventeen assessed. Spot counts with media alone were subtracted. Vertical lines depicting median values are shown. IFN-γ-DP, no. of IFN-γ spots in individuals with positive responses for both IFN-γ and IL-2 ELISPOT assays; IFN-γ-SP, no. of IFN-γ spots in individuals with positive ELISPOT responses for IFN-γ alone; IL-2-DP, no. of IL-2 spots in individuals with positive responses for both IFN-γ and IL-2 ELISPOT assays; IL-2-SP, no. of IL-2 spots in individuals with positive ELISPOT responses for IL-2 alone. (*) P<0.01 vs. IL-2-DP; vs. IL-2-SP and vs. IFN-γ-SP by the Kruskal-Wallis test with Dunn correction. (B) Class I-restricted IFN- γ and IL-2 ELISPOT responses to *trans*-sialidase peptides. Representative positive ELISPOT responses for IFN-γ (grey bars) and IL-2 (white bars) in a single subject are shown. (*) Indicates positive responses, as defined in the Materials and Methods.

More than 75% of infected children had detectable cytokine responses to *T. cruzi*-antigens, with secretion of IFN-γ and IL-2 in nearly 60% of *T. cruzi*-infected children and, approximately 18% with IFN-γ-only responses. IL-2-only responses were not observed in *T. cruzi*-infected children (Table 2). None of the seronegative children born from mothers with positive serology for *T. cruzi* infection had positive ELISPOT responses (Table 2). The number of IFN-γ spots in subjects with concomitant IFN-γ and IL-2 positive ELISPOT responses was higher than the number of IFN-γ spots in IFN-γ-only responders (Figure 1A). Likewise, the former group showed higher spot counts for IFN-γ than IL-2

**Table 2:** *Trypanosoma cruzi*-specific T cell responses in children with Chagas disease and uninfected subjects living in non-endemic areas of Argentina, measured by IFN-γ and IL-2 ELISPOT.

	No. of positive responders/total no. of subjects evaluated (%)	
Positive ELISPOT responses to *T. cruzi* lysate	*T. cruzi*-infected	Uninfected
IFN-γ+IL-2	10/17 (58.8)^A^^, ^ B ^, ^ C	0/15 (0)
IFN-γ-only	3/17 (17.6)^D^	0/15 (0)
IL-2-only	0/17	0/15

A Fisher exact test p = 0.0181 vs. percentage of IFN-γ-only responders in *T. cruzi*-infected subjects.

B Fisher exact test p = 0.0003 vs. percentage of IL-2-only responders in *T. cruzi*-infected subjects.

C Fisher exact test p = 0.0003 vs. percentage of IFN-γ+IL-2 responders in the uninfected group.

D Fisher exact test p = 0.23 vs. percentage of responders in the uninfected group.

**Figure 2:**
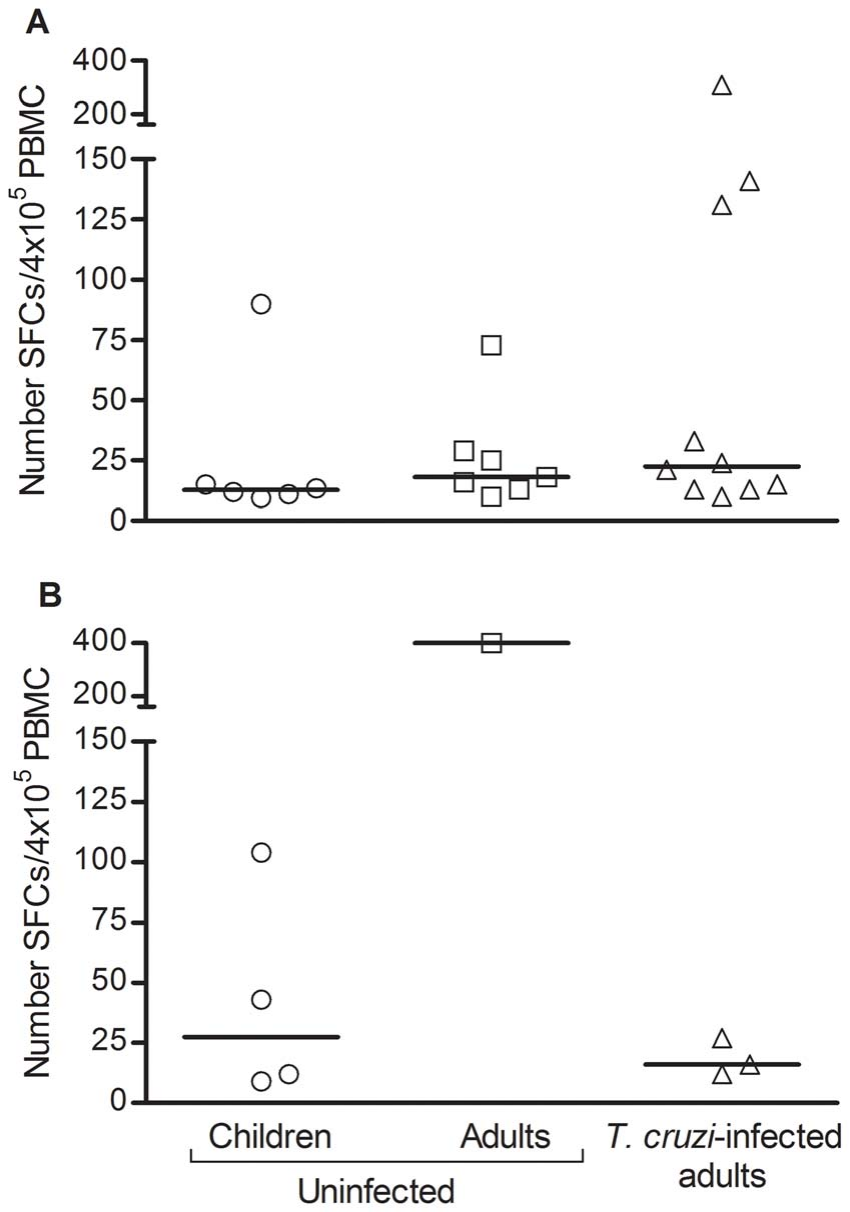
IFN-γ-secreting cells in response to *Flu- and tetanus toxoid-derived antigens* in *uninfected* children, uninfected adults and adults with chronic *T. cruzi*-infection. PBMC from uninfected children (n = 6), uninfected adults (n = 7) and long-term *T.cruzi*-infected adults (n = 10) were seeded at 4×10^5^ cells/well and stimulated with class-I-restricted Flu-derived peptides (A), tetanus toxoid (B) or media alone for 16–20 h. Each symbol represents the mean spot number of triplicate wells for each patient with positive ELISPOT responses, as defined in material and methods. Spot counts with media alone were subtracted. Vertical lines depicting median values are shown. No significant differences were found among groups.

PBMC available from five *T. cruzi*-infected children were also stimulated with peptides encoded by the *trans*-sialidase gene family that bind alleles representative of the 6 most common class I HLA-supertypes, previously shown as targets of CD8^+^ T cell responses in chronically *T. cruzi*-infected adults [7]. Strong IFN-γ ELISPOT responses specific for five out of the six supertype-binding *trans*-sialidase pooled peptides assessed were observed in one out of the five children (Figure 1B). In this same child, strong IL-2 responses to HLA-A3 and HLA-A24-binding peptides were also detected (Figure 1B). In opposition to *T. cruzi*-specific T cell responses, the magnitude of IFN-γ ELISPOT responses to irrelevant antigens, including class-I-restricted Flu peptides and tetanus toxoid, did not differ among uninfected children, uninfected adults and *T. cruzi*-infected adults (Figure 2), supporting the impairment of T cell responses is restricted to *T. cruzi*.

### Cytokine/costimulation coexpression profiles in *T. cruzi*-infected children and adults

**Figure 3:**
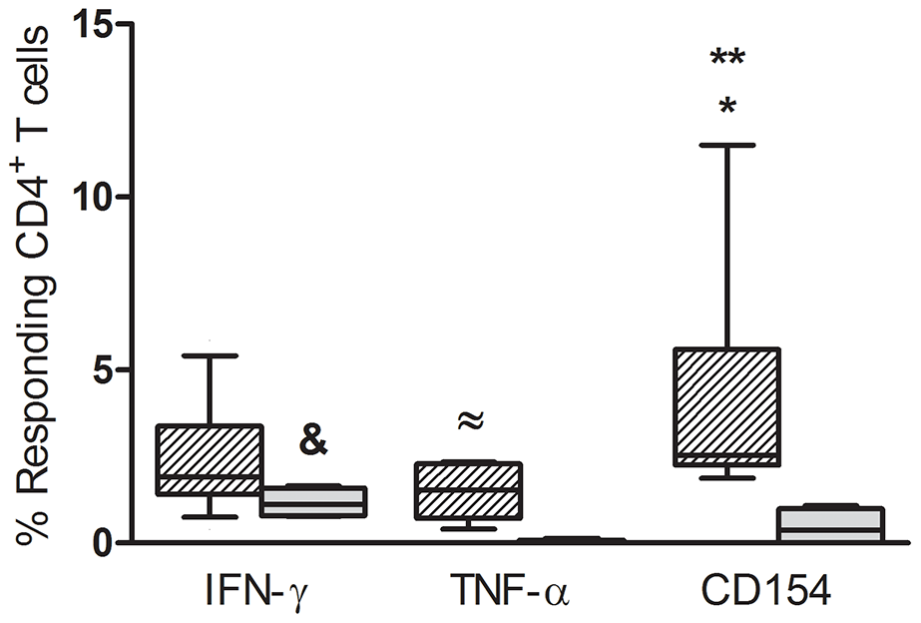
Magnitude of CD4^+^ T cell responses in *T. cruzi*-infected children and adults. Whole blood was stimulated with an amastigote lysate preparation, and antigen-responsive CD4^+^ T cells were measured using an intracellular staining assay for IFN-γ, TNF-α and CD154. The percentage of total responsive CD4^+^ T cells for each individual function in nineteen *T. cruzi*-infected children (hatched columns) and ten adults with chronic *T. cruzi* infection (black columns) is plotted. Boxes and whiskers depicting median and 10th and 90th percentile values are shown. (*) P = 0.03 vs. CD4^+^TNF-α^+^ in *T. cruzi*-infected children and (&) P = 0.03 vs. CD4^+^TNF-α^+^ T cells in *T. cruzi*-infected adults by Kruskal-Wallis test with Dunn correction; (**) P = 0.004 vs. CD4^+^CD154^+^ in *T. cruzi*-infected adults and (≈) P = 0.006 vs. CD4^+^TNF-α^+^ T cells in *T. cruzi*-infected adults by Mann-Whitney U test.

To further determine the quality of *T. cruzi* specific T cell responses earlier in infection, cytokine/co-stimulation co-expression profiles were assessed by intracellular staining following stimulation of whole blood with *T. cruzi* lysate. Since the *T. cruzi* lysate has proved to mainly induce CD4^+^ T cell responses and to a much lesser extent CD8^+^ T cell responses in chronic *T. cruzi*-infections [7], [8], we focused on CD4^+^ T cell responses for flow cytometry assays. The ability of CD4^+^ T cells to produce IFN-γ, TNF-α and CD154 (CD40L) - a marker of costimulatory potential - was evaluated in 19 *T. cruzi*-infected children. For comparison, this functional profile was also assessed in 10 *T. cruzi*-infected adults without evidence of cardiac involvement or with mild cardiac disease. Examining IFN-γ, TNF-α and CD154^+^ individually, CD154 was the most highly expressed in *T. cruzi*-infected children and there was a trend for higher proportions of cells expressing one or more of these three markers in the in the *T. cruzi*-infected children relative to the adults (Figure 3 and Figure S1). IFN-γ-producing CD4^+^ T cells was prevalent among *T. cruzi*-infected adults (Figure 3).

**Figure 4:**
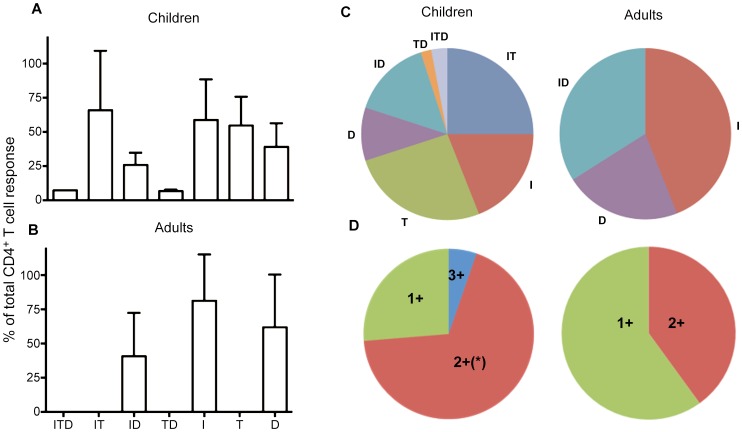
Cytokine/costimulation profile of *T. cruzi*-specific CD4^+^ T cells in short-term and long-term *T. cruzi* infections. PBMC were stimulated with a *T. cruzi* lysate preparation and cytokine/costimulation coexpression profiles with triple (ITD), double (IT, ID, TD) or single (I, T, D) function were determined in nineteen *T. cruzi*-infected children (A) and ten adults with chronic *T. cruzi* infection (B) using the Boolean gating function of FlowJo software. The proportions of CD4^+^ subsets positive for specific cytokines or co-stimulatory molecules were expressed as percentages of total cytokine/costimulatory-positive CD4^+^ T cells. Mean and SD are shown. (C) Summary of the functional composition of the CD4^+^ T cell response. Each slice of the pie represents the fraction of the total response that consists of CD4^+^ T cells positive for the different seven subsets. D) Prevalence of polyfunctional CD4^+^ T cell responses in *T. cruzi*-infected children (left panel) and adults (right panel). Each slice of the pie represents the percent of subjects with one (1+), two (2+) or three (3+) functions. I, IFN-γ; T, TNF-α; D, CD154. ITD, proportion of CD4^+^ T cells with concomitant expression of IFN-γ, TNF-α and CD154; IT, proportion of CD4^+^ T cells with concomitant expression of IFN-γ and TNF-α; ID, proportion of CD4^+^ T cells with concomitant expression of IFN-γ and CD154; TD, proportion of CD4^+^ T cells with concomitant expression of TNF-α and CD154; I, proportion of CD4^+^ T cells with IFN-γ only production; T, proportion of CD4^+^ T cells with TNF-α only production; D, proportion of CD4^+^ T cells with CD154 only expression.

A Boolean gating analysis was then performed to categorize cytokine/CD154 positive cells into seven different subsets consisting of triple, double or single cytokine/CD154-expressing populations. *T. cruzi* antigen responsive CD4^+^ T cells in children with positive serology for *T. cruzi* infection had representatives of all seven possible subsets (range 1–5 subsets per subject) (Figure 4A and 4C). CD4^+^ T cells responding to the lysate exert at least 2 functions in >90% of *T. cruzi*-infected children (Figure 4D). Polyfunctional cytokine-producing cells that simultaneously secrete IFN-γ and TNF-α dominated the CD4^+^ T cell responses in *T. cruzi*-infected children, followed by monofunctional and double expressing IFN-γ^+^CD154^+^ CD4^+^ T cells. TNF-α^+^CD154^+^ and triple expressing IFN-γ^+^TNF-α^+^CD154^+^ cells represented, in average, less than 20% of the total CD4^+^ T cell response to *T. cruzi*-antigens (Figure 4A and 4C). In contrast to children, CD4^+^ T cell responses in infected adults had representatives of only 3 out of the 7 possible subsets (Figure 4B and 4C), with a higher proportion of subjects displaying CD4^+^ T cells with single function than double function and none with all three functions (Figure 4D). In adult patients, monofunctional responses were dominated by IFN-γ-producing T cells followed by CD154^+^ T cells, whereas IFN-γ^+^CD154^+^ cells were predominant among the subsets with double function (Figure 4B).

Monofunctional CD154^+^ followed by monofunctional IFN^+^ T cells and double expressing IFN-γ^+^CD154^+^ T cells showed the highest magnitude of CD4^+^ T cell responses to *T. cruzi* antigens in *T. cruzi*-infected children (Figure S2A). Likewise, monofunctional T cells are also the main contributors to the total magnitude of CD4^+^ T cell responses to the lysate in *T. cruzi*-infected adults, but contrasting with children, T cell responses were enriched in single IFN-γ^+^ rather than single CD154^+^ T cells (Figure S2B).

### CD4^+^ T cell phenotype in *T. cruzi*-infected children

We have previously reported that *T. cruzi*-infected children exhibit increased levels of terminally differentiated CD8^+^ T cells in the overall T cell compartment [9]. In this study, the phenotype of total CD4^+^ T cells was evaluated for the expression of CD45RA, the co-stimulation molecules CD27 and CD28, the lymph node homing receptors CCR7 and CD62L, the IL-7 receptor CD127 and the co-inhibitory receptor killer-cell lectin like receptor G1 (KLRG-1), a marker of the number of TCR-triggering events [21], [22]. Highly differentiated memory T cells (CD45RA^−^CD27^−^CD28^−^ and CD45RA^−^CCR7^−^CD62L^−^) and terminally differentiated effector T cells (CD45RA^+^CCR^−^CD62L^−^) are increased in *T. cruzi*-infected children compared with uninfected controls (Table 3). The increased expression of HLA-DR indicates a high degree of activation of CD4^+^ T cells, while the increased expression of KLRG-1 along with a loss of the IL-7 receptor, suggests sustained antigen exposure of T cells in *T. cruzi*-infected children.

**Table 3:** T cell phenotype of total CD4^+^ T cells in children in the early stages of Chagas disease.

CD4^+^ T cell subset (%)	*T. cruzi*-infected (†)	n	Uninfected (†)	n	P (*)
Total CD4	34 (16–48)	49	32 (14–45)	35	NS
*Naive*					
CD45RA^+^CD27^+^CD28^+^	17 (11–28)	18	21 (6–38)	33	NS
*Early differentiated memory*					
CD45RA^−^CD27^+^CD28^+^	13 (7–36)	18	12 (6–24)	30	NS
*Late differentiated memory*					
CD45RA^−^CD27^−^CD28^−^	1 (0.04–4)	18	0.25 (0–2)	31	0.0004
*CM*					
CD45RA^−^CCR7^+^CD62L^+^	14 (0.54–29)	49	15 (2–52)	33	NS
*EM*					
CD45RA^−^CCR7^−^CD62L^−^	12 (3–26)	49	8 (0–19)	33	0.001
*TE*					
CD45RA^+^CCR7^−^CD62L^−^	1.42 (0.2–14)	49	0.5 (0–5)	31	0.0001
*Activation/Antigen exposure*					
HLA-DR^+^	11.5 (1.8–35.5)	43	2 (0.75–14)	9	0.0006
IL7R^+^	91 (68–98)	49	94.3 (88–98)	29	0.0004
CD45RA^−^KLRG1^+^IL-7R^−^	0.6 (0.06–1.7)	44	0.3 (0.02–1.1)	30	0.006

Note. (†) Data are expressed as the median (range) percentage of total CD4^+^ T lymphocytes.

For each phenotype. (*) Mann-Whitney U-test between *T. cruzi*-infected and uninfected subjects; NS, no significant different; CM, central memory; EM, effector memory; TE, terminally differentiated effector cells.

## Discussion

T cell deficiencies resulting from exhaustion include a hierarchical loss of effector functions along with the expression of inhibitory receptors, and failure to exhibit antigen-independent homeostatic proliferation [5], [23], [24]. Although, this phenomenon was initially described for chronic viral infections, recent studies have shown that this process can also occur in protozoan diseases [6], [25]. We have previously provided several pieces of evidence of immune exhaustion in adults with chronic *T. cruzi*-infection [8], [26]–[29].

In this study, we report for the first time that *T. cruzi*-infected children in early stages of *T. cruzi* infection maintained polyfunctional CD4^+^ T cells responsive to *T. cruzi* antigens in their circulation. More than ninety percent of *T. cruzi*-infected children displayed responsive CD4^+^ T cells with double or triple functions. These findings are in sharp contrast to the profile observed in a group of adults with chronic *T. cruzi*-infections, in which most subjects showed monofunctional *T. cruzi*-specific responses enriched in single IFN-γ^+^ T cells with absence of TNF-α^+^-producing T cells. Other authors had also reported that children in the early phase of chronic *T. cruzi* infection exhibit IFN-γ^+^- and TNF-α^+^-secreting CD4^+^ T cells in response to *T. cruzi* antigens [10], [11].

The analysis of IFN-γ ELISPOT responses to the lysate and to *trans*-sialidase-derived class I peptides in *T. cruzi*-infected children also revealed that not only were the proportion with polyfunctional responses higher relative to infected adults, but that the magnitude of IFN-γ responses in these positive children was higher by 5-fold than previously reported in chronically *T. cruzi*-infected adults (i.e. median number of IFN-γ spots/4×10^5^ PBMC in children = 220 vs. 52 spots evaluated in 150 subjects in the G0 group), [Laucella], [ unpublished data, 7,8]. Of note, the one single child responsive to *trans*-sialidase-derived CD8^+^ target peptides showed a stronger and broader response compared with our previous findings in adults (i.e. median number of IFN-γ spots/4×10^5^ in this child = 87 spots, with five out of the six supertype-binding *trans*-sialidase pooled peptides recognized in average vs. 19 spots in 25 *T. cruzi*-infected adults, with two out of six pooled peptides recognized in average) [7]. The differences are even more dramatic when comparing *T. cruzi*-infected children with adults exhibiting severe cardiomyopathy who mostly showed negative T cell responses against *T. cruzi* antigens [7], [8]. Recent studies have also shown that individuals with severe cutaneous leishmaniasis have reduced CD8^+^ T cell numbers with the ability to produce IFN-γ and IL-2 [30].

Multifunctional T cells are optimized for effector function, exhibiting a higher secretion of IFN-γ on a per-cell basis, more efficient killing, and IL-2-mediated expansion of T cells in an autocrine or paracrine manner [31]. In agreement with these findings, we observed a higher level of IFN-γ production in *T. cruzi*-infected children displaying both IFN^+^ and IL-2^+^ ELISPOT responses compared with those with single IFN-γ-producing T cells. It is likely that the maintenance of polyfunctional T cell responses provides more efficient control of *T. cruzi* infection and limits peripheral tissue damage. Indeed, most *T. cruzi*-infected children have not developed cardiac disease and previous studies from our group have shown higher frequencies of IFN-γ-producing T cells and higher magnitude of these responses in subjects with less severe forms of the disease compared with patients with severe cardiomypathy [7], [8], [29]. Of note, seropositive children who were born and lived for some years in areas endemic for *T. cruzi* infection showed higher electrocardiographic abnormalities not related to Chagas disease compared to children born in non-endemic areas. This is likely due to co-morbidities and poorer socioeconomic conditions in rural areas. In this respect, we have shown that socioeconomic conditions had a significant influence on the progression of chronic Chagas disease which was independent of antiparasitic treatment and clinic characteristics [32]. Nevertheless, the magnitude and quality of *T. cruzi*-specific T cell responses were not different between seropositive children born in endemic areas and those born in non-endemic areas, who are presumed to have been infected congenitally.

In *T. cruzi* infection, parasite persistence for decades, might drive a process of immune exhaustion. A broader range (relative to adults) of *T. cruzi*-specific immune phenotypes are present in *T. cruzi*-infected children – from “adult-like” monofunctional CD4^+^ T cell subsets to T cells exhibiting up to 3 functional properties. Thus, some *T. cruzi*-infected children display a T cell profile similar to that of adult patients. This is not unexpected since some children will likely have infections of >10 years. The increased frequencies of total effector/effector memory and activated CD4^+^ T cells in *T. cruzi*-infected children observed herein suggest a heightened state of immune activation as previously suggested in other studies [9], [33], [34] In contrast to the unaltered levels of naïve CD4^+^ T cells shown herein, previous studies by our group in a similar patient population of children demonstrated a significant decrease in the levels of naïve CD8^+^ T cells compared to uninfected children [9]. Likewise, Vitelli-Avelar reported decreased levels of CD62L^+^CD4^+^ T cells but lower frequencies of CD4^+^HLA-DR^+^ T cells along with unaltered levels of CD4^+^CD28^+^ T cells [35] in children in the indeterminate phase of the infection. Adults with chronic *T. cruzi* infection also show increased levels of activated, late differentiated memory and effector T cells, while naïve T cells are diminished as disease becomes more severe [26], [27], [29], [36]. A crucial question remains whether the heterogeneity in the functional and phenotypic profile is a predictor of different disease outcome in these patients.

Children have not only shorter term infections but are also younger. Aging of the immune system in elderly subjects (generally older than 65 years of age), known as immunosenescence [37], is mainly associated to poor responsiveness to new pathogens and reduced efficacy of vaccination-induced protection against infection, while established memory immune responses to previously encountered pathogens are much less affected unless it is additionally stressed by chronic infections or autoimmune diseases [38], [39]. Several studies have shown accelerated immune aging in HIV-infected subjects and subjects chronically infected with cytomegalovirus [40]. In this study, chronically

*T. cruzi*-infected subjects are younger than 60 years, in average, and there were not differences in the magnitude of IFN-γ ELISPOT to irrelevant antigens like toxoid tetanus and Flu among uninfected children, uninfected adults *T. cruzi*-infected adults,. Of note, in previous studies, we did not found any association between the magnitude of T cell responses specific for *T. cruzi* and age in a cohort of chronically *T. cruzi*-infected subjects in an age range of between 22 and 70 years [7], [8]. Furthermore, CD4+ T cells responsive to *T. cruzi*-antigens in *T. cruzi*-infected adults display high expression of the inhibitory receptor CTLA-4 which is a feature of exhausted T cells [41], while few T cells responsive to tetanus and diphtheria toxoids expressed CTLA-4 [29], supporting that impairment in T cell responses in *T. cruzi*-infected adults was confined to those specific for T. cruzi. The phenotype of the overall T cell compartment with an expansion of end-differentiated effector T cells and loss of naïve T cells in *T. cruzi*-infected children and adults compared with age-matched controls suggest that chronic *T. cruzi* infection might accelerate immune aging along with the induction of T cell exhaustion.

The quality of the immune response might also be a factor in the higher cure rates following drug treatment observed in children relative to adults [42]. It had been proposed that IFN-γ production might promote the effectiveness of chemotherapy in patients with chronic *T. cruzi* infections [12], [43]. All the children included in this study were treated with benznidazole, and are currently under follow-up. It will be of interest to address whether the response to therapy is associated with the qualitative and quantitative characteristics of the anti-*T.cruzi* T cell response prior to treatment.

## Supporting Information

Figure S1 ***T. cruzi*** **–specific CD4^+^ T cell profile in children and adults with Chagas disease.** Co-expression profiles of CD4^+^ T cells with the ability to produce IFN-γ, TNF-α, or to express the co-stimulatory molecule CD154 were measured by flow cytometry after 16–20 hs stimulation of whole blood with a *T. cruzi* lysate preparation. (A) Representative dot plots from one *T. cruzi*-infected child and one adult with chronic *T. cruzi* infection are shown. The numbers in the upper right quadrant indicate the percentage of CD4^+^IFN-γ^+^, CD4^+^TNF-α^+^ or CD4^+^ CD154^+^ T lymphocytes responsive to the lysate. (B) Representative dot blot of IFN-γ and TNF-α co-production by CD4^+^ T cells from the same patients showed in A. The numbers in the upper right quadrant indicate the percentage of CD4^+^IFN-γ^+^TNF-α^+^ T cells, while the numbers in the upper left represent the percentage of CD4^+^IFN-γ^+^TNF-α^−^ T lymphocytes on total CD4^+^ T cells.
(TIF)

Figure S2 ***T. cruzi*** **-specific CD4^+^ T cell responses in children and adults with Chagas disease.** PBMC were stimulated with a *T. cruzi* lysate preparation and stained for IFN-γ, TNF-α and CD154. Triple, double or single functional profiles were determined by FlowJo Boolean gating analysis in nineteen *T. cruzi*-infected children (A) and ten *T. cruzi*-infected adults (B). Data are presented as the percentage of responding CD4^+^ T cells in each subset. Boxes depicting median and 5th and 95th percentile values are shown. I, IFN-γ; T, TNF-α; D, CD154. (**) P<0.01 vs. ITD and TD among *T. cruzi*-infected children; (^&^) P<0.05 vs. IT and T among *T. cruzi*-infected children; (*) P<0.05 vs. ITD, IT, TD and T among *T. cruzi*-infected adults, by the Kruskal-Wallis test with Dunn correction.
(TIF)
