# Supplementary figures and images for: Polyfunctional T Cell Responses in Children in Early Stages of Chronic Trypanosoma cruzi Infection Contrast with Monofunctional Responses of Long-term Infected Adults
Source: PLoS Negl Trop Dis. 2013 Dec 12;7(12):e2575. doi: 10.1371/journal.pntd.0002575 (PMC3861186; doi:10.1371/journal.pntd.0002575)

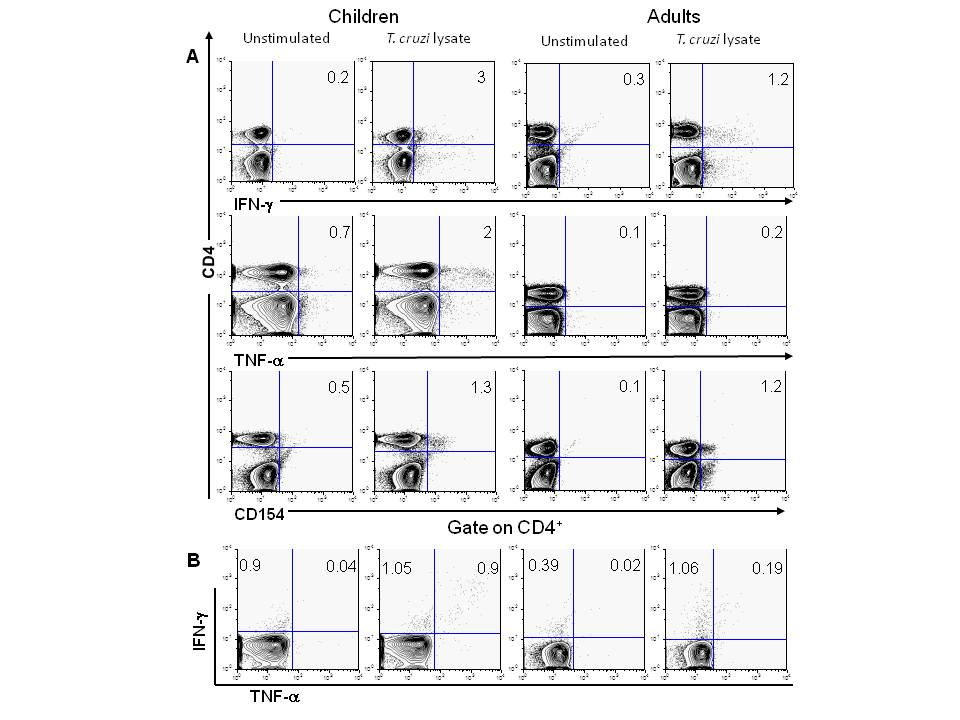

Supplement: Figure S1 — T. cruzi –specific CD4+ T cell profile in children and adults with Chagas disease. Co-expression profiles of CD4+ T cells with the ability to produce IFN-γ, TNF-α, or to express the co-stimulatory molecule CD154 were measured by flow cytometry after 16–20 hs stimulation of whole blood with a T. cruzi lysate preparation. (A) Representative dot plots from one T. cruzi-infected child and one adult with chronic T. cruzi infection are shown. The numbers in the upper right quadrant indicate the percentage of CD4+IFN-γ+, CD4+TNF-α+ or CD4+ CD154+ T lymphocytes responsive to the lysate. (B) Representative dot blot of IFN-γ and TNF-α co-production by CD4+ T cells from the same patients showed in A. The numbers in the upper right quadrant indicate the percentage of CD4+IFN-γ+TNF-α+ T cells, while the numbers in the upper left represent the percentage of CD4+IFN-γ+TNF-α− T lymphocytes on total CD4+ T cells. (TIF) [file pntd.0002575.s001.tif]

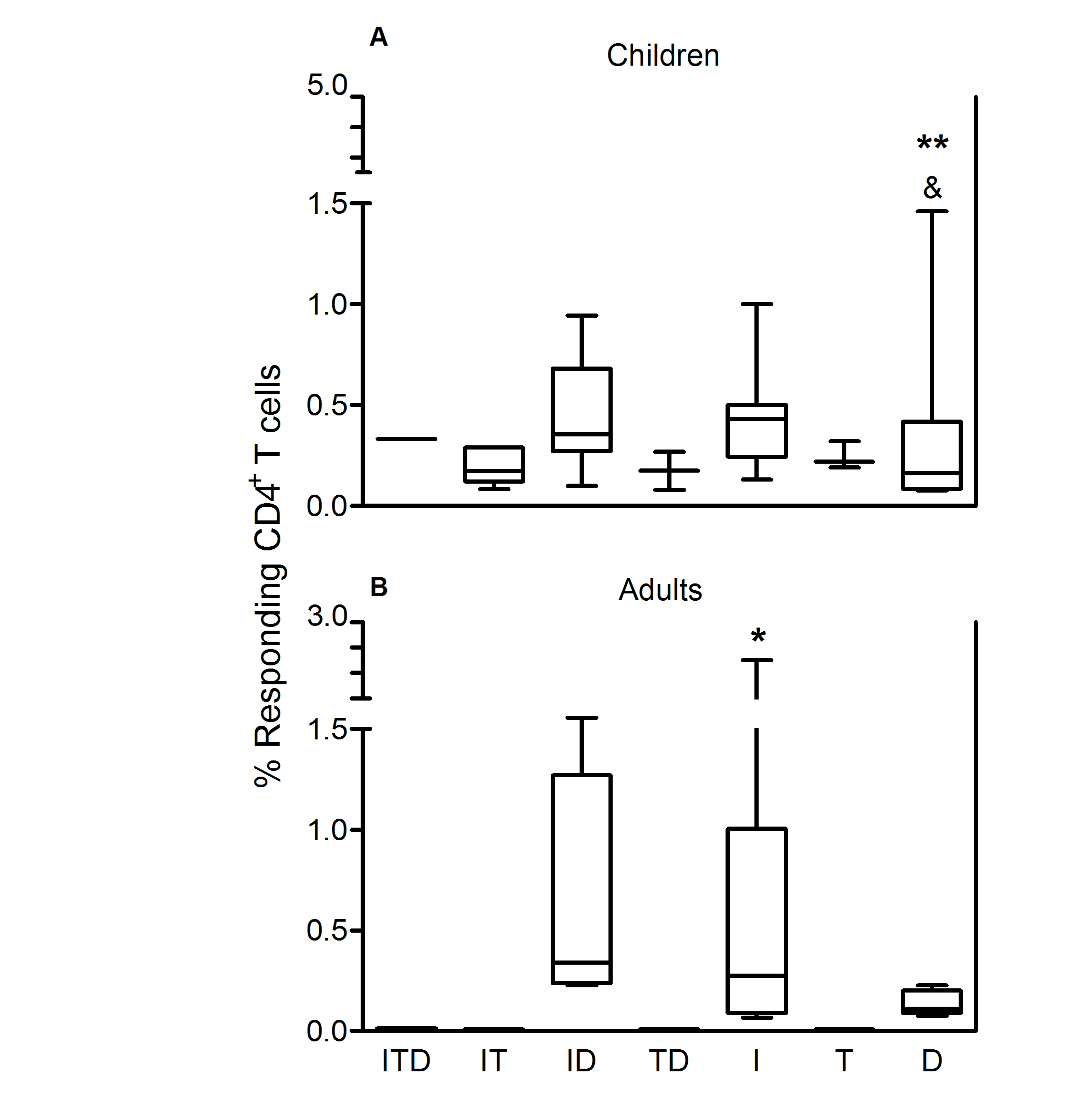

Supplement: Figure S2 — T. cruzi -specific CD4+ T cell responses in children and adults with Chagas disease. PBMC were stimulated with a T. cruzi lysate preparation and stained for IFN-γ, TNF-α and CD154. Triple, double or single functional profiles were determined by FlowJo Boolean gating analysis in nineteen T. cruzi-infected children (A) and ten T. cruzi-infected adults (B). Data are presented as the percentage of responding CD4+ T cells in each subset. Boxes depicting median and 5th and 95th percentile values are shown. I, IFN-γ; T, TNF-α; D, CD154. (**) P<0.01 vs. ITD and TD among T. cruzi-infected children; (&) P<0.05 vs. IT and T among T. cruzi-infected children; (*) P<0.05 vs. ITD, IT, TD and T among T. cruzi-infected adults, by the Kruskal-Wallis test with Dunn correction. (TIF) [file pntd.0002575.s002.tif]
